# Are in Person and Telephone Interviews Equivalent Modes of Administrating the CAT, the FACIT-FS and the SGRQ in People With COPD?

**DOI:** 10.3389/fresc.2021.729190

**Published:** 2021-10-25

**Authors:** Vânia Rocha, Cristina Jácome, Vitória Martins, Alda Marques

**Affiliations:** ^1^Lab3R-Respiratory Research and Rehabilitation Laboratory, School of Health Sciences (ESSUA), University of Aveiro, Aveiro, Portugal; ^2^iBiMED-Institute of Biomedicine, University of Aveiro, Aveiro, Portugal; ^3^Center for Health Technology and Services Research (CINTESIS), Faculty of Medicine, University of Porto (FMUP), Porto, Portugal; ^4^Department of Community Medicine, Information and Health Decision Sciences (MEDCIDS), Faculty of Medicine, University of Porto, Porto, Portugal; ^5^Pulmonology Department, Hospital Distrital da Figueira da Foz, Figueira da Foz, Portugal

**Keywords:** chronic obstructive pulmonary disease, assessment and monitoring, COPD assessment test, functional assessment of chronic illness-fatigue subscale, St. George's respiratory questionnaire, telephone interview, patient-reported outcome measures

## Abstract

**Background:** The COVID-19 pandemic brought numerous challenges, namely in routine assessment of people with chronic obstructive pulmonary disease (COPD). The COPD Assessment Test (CAT), the Functional Assessment of Chronic Illness-Fatigue-Subscale (FACIT-FS) and the St. George's respiratory questionnaire (SGRQ) are important patient-reported outcome measures used to assess people with COPD, but its face-to-face application has been compromised. The telephone interview offers a simple and effective alternative, yet uncertainty regarding its equivalence remains. This study aimed to establish the reliability and validity of the CAT, the FACIT-FS and the SGRQ administered by telephone interview in people with COPD.

**Methods:** Data from an observational prospective study including people with COPD were analyzed. Participants answered to the CAT, FACIT-FS and SGRQ questionnaires in person and by telephone, with a maximum interval of 48-h. Participants were randomly selected to answer first to the in-person questionnaire followed by telephone or vice versa. Reliability measures included internal consistency with Cronbach's alpha, test-retest reliability with the intraclass correlation coefficient (ICC_2,1_), test-retest measurement error with the standard error of measurement (SEM) and agreement with the Bland and Altman 95% limits of agreement. Validity was assessed with the Spearman correlation (rho).

**Results:** Fifty-five people with COPD (44 men; 68.1 ± 7.9 years; FEV_1_: 59.1 ± 20.3% predicted) were included. Similar internal consistency was observed between in person vs. telephone interview for the CAT (0.82 vs. 0.84), the FACIT-FS (0.83 vs. 0.84) and the SGRQ (0.92 vs. 0.93). Test-retest reliability was excellent, with an ICC_2,1_ of 0.77 (95% CI: 0.65; 0.86), 0.86 (95% CI: 0.77; 0.92) and 0.94 (95% CI: 0.90; 0.96) for the CAT, FACIT-FS and SGRQ total scores, respectively. The SEM showed a low level of associated measurement error and the Bland and Altman plots illustrated a good level of agreement between both modes of administration, with no evidence of systematic bias. Robust positive correlations (rho 0.87–0.94, *p* < 0.001) were found for the CAT, FACIT-FS and SGRQ total scores applied by both methods.

**Conclusion:** The telephonic administration of the CAT, the FACIT-FS and the SGRQ are a valid and reliable alternative approach to in person interviews for monitoring symptoms and health-related quality of life in people with COPD. The telephone might be an important add-on for personalized assessment and management of COPD thru remote monitoring.

## Introduction

Chronic obstructive pulmonary disease (COPD) is a major worldwide health problem due to its high prevalence (about 10% of the adult population), rising mortality and huge burden (1–3). Most of the burden is driven by the impact of COPD symptoms on individuals' daily life (4–6). A symptom-centered treatment approach might be key to improve quality of life, health status and prognosis of people with COPD (4–6) by optimizing symptom control and reducing future risks, such as acute exacerbations, mortality, comorbidities and the long-term consequences of disease (7).

The COVID-19 pandemic impacted patient care significantly (2, 8). The main reason was the reduction in face-to-face appointments which severely limited routine assessments to monitor patients' health status and the delivery of personalized interventions, such as pulmonary rehabilitation (2, 8). Patients' health status is commonly assessed using clinical examination, physical measures and self-reported questionnaires on symptoms and health-related quality of life (HRQoL). While it is understandable that clinical examination and assessment using physical measures can be difficult outside scheduled face-to-face appointments, the same may not be true for self-reported questionnaires. In fact, the routine application of valid and reliable patient-reported outcome measures (PROMS) assessing COPD symptoms and daily functioning is important to prevent and identify complications early (1, 9–11). It also allows optimisation of outcomes for each person (10, 11) thus, should be maintained even in challenging times, with innovative approaches (12, 13).

Evidence suggests that some healthcare can be provided remotely, for instance, by gathering information on symptoms and health status providing education for disease self-management and counseling, e.g., on smoking cessation. Specifically, in the context of the pandemic, multiple actions were driven to implement remote services, including teleconsultation of people with COPD (14–16) and telerehabilitation (8, 17, 18). Therefore, alternative methods to maintain routine assessment of this population and simultaneously reduce social interactions as a way to prevent SARS-CoV-2 propagation are important and deserve to be further explored (8, 17).

Technologies of information and communication, namely the telephone, offer a simple and effective alternative to paper-forms to keep monitoring these individuals (17, 19). Evidence has shown that telephone administration of some health instruments for symptoms control (20) and measurement of HRQoL (21, 22) is comparable to the in-person application. However, the psychometric properties of specific questionnaires to people with COPD still need to be explored; specifically, the reliability and validity of the telephone administration of the Functional Assessment of Chronic Illness-Fatigue-Subscale (FACIT-FS) and the St. George's Respiratory Questionnaire (SGRQ) have never been tested and only one study assessed these properties in the COPD Assessment Test (CAT) (23). Therefore, the equivalence of assessing PROMs, namely the CAT, the FACIT-FS and the SGRQ, by telephone in comparison to paper-forms in people with COPD, remains uncertain.

This information will be fundamental for rethinking alternative models of remote assessment and/or follow-up of people with COPD, both in clinical and research settings, in a context where social interactions should be (e.g., due to health reasons) or are (e.g., due to geographical constraints) reduced, but individuals' continuity of care remains essential (8, 24). Therefore, this study aimed to establish the reliability and validity of the CAT, FACIT-FS and SGRQ administered by telephone interview in people with COPD.

## Methods

### Study Design

This study was conducted between July 2020 and April 2021, as part of an observational prospective study (PRIME - PTDC/SAU-SER/28806/2017) aiming to conduct pulmonary rehabilitation and follow-up participants with COPD over a 6-months period. The following Ethics Committees (Centro Hospitalar do Médio Ave reference 09/2016 and 10/2018; Unidade Local de Saúde de Matosinhos reference 10/CES/JAS 17/02/2017 and 73/CE/JAS 12/10/2018; Centro Hospitalar Baixo Vouga reference 777638 and 086892; Hospital Distrital da Figueira da Foz reference 1807/2017 and 27/05/2019; Administração Regional de Saúde do Centro reference 64/2016 and 85/2018) approved the study.

This study followed the guidelines on measurement properties for patient-reported outcomes of the COnsensus-based Standards for selection of health status Measurement Instruments (COSMIN) initiative (25) and is reported according to the Strengthening the Reporting of Observational Studies in Epidemiology (STROBE) guidelines (26).

### Participants

People with COPD living in the community were recruited by clinicians/pulmonologists at two hospitals (Centro Hospitalar Baixo Vouga and Hospital Distrital da Figueira da Foz) during their routine appointments. Individuals were eligible if diagnosed with COPD (1) and clinically stable for 1 month prior to the study [i.e., no hospital admissions or exacerbations, nor changes in medication, according to Global Initiative for Chronic Obstructive Lung Disease (GOLD) (1)]. Exclusion criteria included the presence of other respiratory diseases or significant cardiovascular, neurologic, or musculoskeletal disease that precluded their participation in the study. Eligible participants were invited to participate in a 12-weeks pulmonary rehabilitation programme which includes supervised exercise training twice per week, education and psychosocial support every 2 weeks (27) and/or to have their health status monitored monthly over a 6-months period. More detailed information on the structure of the pulmonary rehabilitation programme is available elsewhere (28). Before enrolment and data collection, written and verbal description of the study was provided to all participants and the written informed consent was obtained.

### Data Collection

Sociodemographic (age, sex and educational level), anthropometric (height and weight to compute body mass index-BMI) and clinical data (smoking habits, baseline dyspnoea, comorbidities and number of acute exacerbations and hospitalisations in the preceding year) were first collected using a structured questionnaire administered during a baseline face-to-face visit. Lung function values were obtained from participants' medical records and used to establish the severity of airway obstruction according to the GOLD report (1).

Participants' educational level was measured as completed years of schooling and classified into three categories according to the International Standard Classification of Education (ISCED) (29): ≤ 4 years (ISCED 0–1); 5–9 years (ISCED 2); ≥10 years (ISCED 3–8), following current Portuguese study cycles. The smoking status were classified as never, former and current smokers and the pack-years were also computed by multiplying each pack of 20 cigarettes smoked per day by the number of years the person has smoked.

The modified British medical research council (mMRC) questionnaire was used to assess activity-related dyspnoea and scored from 0 (no trouble with breathlessness) to 4 (too breathless to leave the house) (30). The severity of comorbid diseases was scored according to Charlson Comorbidity Index (CCI) (i.e., mild: 1–2; moderate: 3–4; severe: ≥5 scores) (31).

Participants were then asked to answer the CAT, the FACIT-FS and the SGRQ. All questionnaires were administered twice, in-person and by telephone by an interviewer, with a maximum interval of 48-h between the two modes of administration. In person administration occurred during evaluations at the Respiratory Research and Rehabilitation Laboratory (Lab3R) or during a home visit for follow-up assessments. Participants were randomly selected to answer first to the in-person interview followed by telephone interview or vice versa, to ensure that participants had the same opportunity of answering either formats in the first assessment. Some participants had never answered these instruments previously, and others might have answered them in previous assessments occurring at least 3-months ago, which reduced the possibility of a learning effect. A well-trained physiotherapist on COPD assessment and management conducted all the telephone and the in-person interviews. Participants and the physiotherapist were blinded to the answers/scores of the instruments in their first application.

### Instruments

The CAT is a brief and simple questionnaire for assessing and monitoring COPD (32, 33). It consists of eight items covering the most burdensome symptoms and limitations of COPD, for instance cough, phlegm, chest tightness, breathlessness, activity limitations, confidence, sleep, and energy (32, 33). The score for each question ranges from zero to five, and the total score ranges from zero to 40, with higher scores indicating worse health status. A cut-off ≥10 points is associated with a considerable impact of the disease in individuals' daily life (34). The CAT is also recommended to classify participants according to the ABCD assessment tool (1).

The FACIT-FS evaluates tiredness, weakness and difficulty in handling daily activities due to fatigue over the prior 7 days (35, 36). This scale is a multidimensional 13-item questionnaire in which items are classified according to a five-point Likert scale from “not at all” to “very much” (36). The scores range from zero to 52, with higher scores indicating less fatigue (36). Individuals scoring below the cut-off of 43 points were considered to have clinically relevant fatigue (37).

The SGRQ is a disease-specific instrument measuring HRQoL in people with chronic lung disease (38). The questionnaire has three domains: symptoms, activities, and impact (38). Each domain and the total questionnaire is scored from zero (no impairment) to 100 (maximum impairment), with lower scores representing a better HRQoL (39). A cut-off ≥ 25 points is indicative of the disease impact on HRQoL.

### Psychometric Properties

The psychometric properties assessed for the CAT, the FACIT-FS and the SGRQ were reliability and validity.

Reliability is the ability to reproduce a consistent result in time and space, or from different observers, presenting aspects of coherence, stability, equivalence and homogeneity (25, 40). Reliability estimates may be used to evaluate the equivalence of a set of items from the same test (internal consistency) and the stability of measures administered at different times to the same individuals or using the same standard (test–retest reliability) (40). Relative and absolute reliability are complementary methods and thus, should both be tested (25). Reliability coefficients range from zero to one, with higher coefficients indicating higher levels of reliability (40).

Validity is defined as the extent to which an instrument measures what it intends to measure (25, 40). Specifically, for establishing concurrent validity, scores of an instrument are correlated with scores of other measure which assesses the same construct and in the same subjects (40). Validity requires that an instrument is reliable, but an instrument can be reliable without being valid (40).

### Statistical Analyses

Descriptive statistics were used to characterize the sample. Normal distribution of the CAT, FACIT-FS and SGRQ scores was tested using the Kolmogorov–Smirnov test. Differences between the questionnaires scores applied in person vs. by telephone were tested using the Wilcoxon signed rank test.

The internal consistency was assessed using the Cronbach's alpha and a value higher than 0.70 was considered an acceptable level of consistency (41). Relative and absolute reliability were established. Relative reliability in test-retest was explored with the Intraclass Correlation Coefficient (ICC) equation (1, 2) and the respective 95% confidence intervals (95%CI) (42). The ICC was interpreted as excellent (>0.75), moderate to good (0.4–0.75) or poor (<0.4) (43). Absolute reliability, i.e., the agreement between the scores obtained in person *vs*. by telephone interview, was tested with the Bland and Altman method and the standard error of measurement (SEM). The first was used for visual judgement of how well the scores obtained by the two modes of administration agreed (44) and the second indicated the extent to which the scores varied on repeated measurements, providing a value for measurement error in the same units as the measurement itself (42). The SEM formula is: SEM = SD√ (1-ICC), where SD is the standard deviation of the scores obtained from the total sample of each scale.

The concurrent validity of the questionnaires applied in person and by telephone was assessed with the Spearman correlation coefficient (rho). This coefficient was interpreted as strong (≥0.70), moderate (0.30–0.70) or weak (≤0.30) (45).

A sample size of at least 50 participants are needed to test the psychometric properties of PROMs, although a sample size of 100 participants is desirable according to the COSMIN initiative (25).

All statistical analyses were conducted using IBM SPSS Statistics, version 24 (IBM Corporation, Armonk, NY, USA). The level of significance was set at *p*-value (p) ≤ 0.05.

## Results

### Participants

Fifty-nine people with COPD were invited to participate in the study. Four individuals were excluded due to the presence of significant cognitive (*n* = 1) and musculoskeletal (*n* = 1) impairment, and absence of a confirmed diagnosis of COPD (*n* = 2). Table 1 shows the baseline characteristics of the 55 participants included in the analysis. Participants' mean age was 68.1 (± 7.9) years, 80% were male and presented a mean BMI of 27.3 (± 4.6) kg/m^2^. About 38.2% of participants had less or equal to four years of education (ISCED 0–1), were mostly former smokers (80%), presented a median mMRC grade of 2 [1; 3] and mild to moderate airflow limitation (65.4%). No significant differences were observed in the average scores of the CAT [11.3 (± 4.6) vs. 10.6 (± 7.4), *p* = 0.191], the FACIT-FS [41.4 (± 8.1) vs. 42.6 (± 7.0), *p* = 0.058] and the SGRQ [31.7 (± 20.1) vs. 32.1 (± 20.9), *p* = 0.459] assessed by in person *vs*. telephone interview (Table 2).

**Table 1:** Baseline characteristics of people with chronic obstructive pulmonary disease (*n* = 55).

	**Total (*n* = 55)**
**Age, years**	68.1 (7.9)
**Male**, ***n*** **(%)**	44 (80.0)
**BMI (kg/m** ^**2**^ **)**	27.3 (4.6)
**Educational level**, ***n*** **(%)**	
≤ 4 years of education (ISCED 0–1)	21 (38.2)
5–9 years of education (ISCED 2)	16 (29.1)
>9 years of education (ISCED 3–8)	18 (32.7)
**Smoking status**, ***n*** **(%)**	
Never	7 (12.7)
Former	44 (80.0)
Current	4 (7.3)
Pack-years, median [Q25; Q75]	39.0 [11.8; 80.0]
mMRC, median [Q25; Q75]	2 [1;3]
**Pulmonary function**	
FEV1, %predicted	59.1 (20.3)
FVC, %predicted	85.3 (21.0)
FEV1/FVC	53.4 (11.3)
**GOLD grades**, ***n*** **(%)**
1–2	34 (65.4)
3–4	18 (34.6)
**Burden of disease**, ***n*** **(%)**	
**GOLD groups (CAT)**
A–B	50 (90.9)
C–D	5 (9.1)
**CCI**
Mild (1–2 points)	9 (16.4)
Moderate (3–4 points)	36 (65.5)
Severe (≥5 points)	10 (18.2)

*Values are shown as mean (standard deviation) unless otherwise indicated. BMI, body mass index; ISCED, International Standard Classification of Education; CCI, Charlson Comorbidity Index; mMRC, modified medical research council questionnaire FEV_1_, forced expiratory volume in 1 s; FVC, forced vital capacity; GOLD, Global Initiative on Obstructive Lung Disease*.

**Table 2:** The COPD Assessment Test (CAT), the Functional Assessment of Chronic Illness-Fatigue-Subscale (FACIT-FS) and the St. George's Respiratory Questionnaire (SGRQ) scores applied in person and by telephone interview to people with chronic obstructive pulmonary disease (*n* = 55).

	**In person interview**	**Telephone interview**	***p*-value^a^**
**CAT**, total score	11.3 (8.3)	10.6 (7.4)	0.191
**FACIT-FS**, total score	41.4 (8.1)	42.6 (7.0)	0.058
**SGRQ**, scores			
Symptoms	37.0 (20.1)	37.7 (20.4)	0.612
Activity	45.9 (27.2)	48.1 (29.6)	0.056
Impact	22.2 (19.4)	21.4 (19.2)	0.525
Total	31.7 (20.1)	32.1 (20.9)	0.459

*Values are shown as mean (standard deviation)*;

a *p-value for the difference using the Wilcoxon signed rank test*.

### Reliability

A similar and strong internal consistency was observed between the telephone vs. in person interview for the CAT (0.82 vs. 0.84), the FACIT-FS (0.83 vs. 0.84) and the SGRQ (0.92 vs. 0.93) (Table 3).

**Table 3:** Reliability of the COPD Assessment Test (CAT), the Functional Assessment of Chronic Illness-Fatigue-Subscale (FACIT-FS), St. George's Respiratory Questionnaire (SGRQ) applied in person and by telephone interview to people with chronic obstructive pulmonary disease (*n* = 55).

	**Internal consistency** **Cronbach's alpha**		**Test-retest reliability ICC _**(2, 1)**_ (95% CI)**	Test-retest measurement error EM^*^(95% CI)
	**In person interview**	**Telephone interview**		
CAT	0.82	0.84	0.77 (0.65; 0.86)	3.54 (3.69; 17.59)
FACIT-FS	0.83	0.84	0.86 (0.77; 0.92)	2.61 (37.47; 47.73)
SGRQ	0.92	0.9-3		
Symptoms	–	–	0.83 (0.73; 0.90)	8.41 (21.25; 54.23)
Activity	–	–	0.94 (0.90; 0.97)	7.24 (33.93; 62.31)
Impact	–	–	0.88 (0.81; 0.93)	6.66 (8.30; 34.42)
Total	–	–	0.94 (0.90; 0.96)	5.13 (22.03; 42.13)

*Values are shown as mean (standard deviation) unless otherwise indicated. ICC, intraclass correlation coefficient; CI, confidence intervals; SEM, standard error of measurement*:

* *SEM calculated with standard deviation of the phone version*.

Excellent test-retest reliability was found for total scores of the CAT [ICC: 0.77 (95% CI: 0.65; 0.86)], the FACIT-FS [ICC: 0.86 (95% CI: 0.77; 0.92)] and the SGRQ [ICC: 0.94 (95% CI: 0.90; 0.96)], as well as for the scores of each SGRQ domain (Table 3). A reduced test-retest measurement error was found among the three questionnaires, ranging between 2.61 in FACIT-FS to 8.41 in SGRQ symptoms subscale (Table 3).

**Figure 1:**
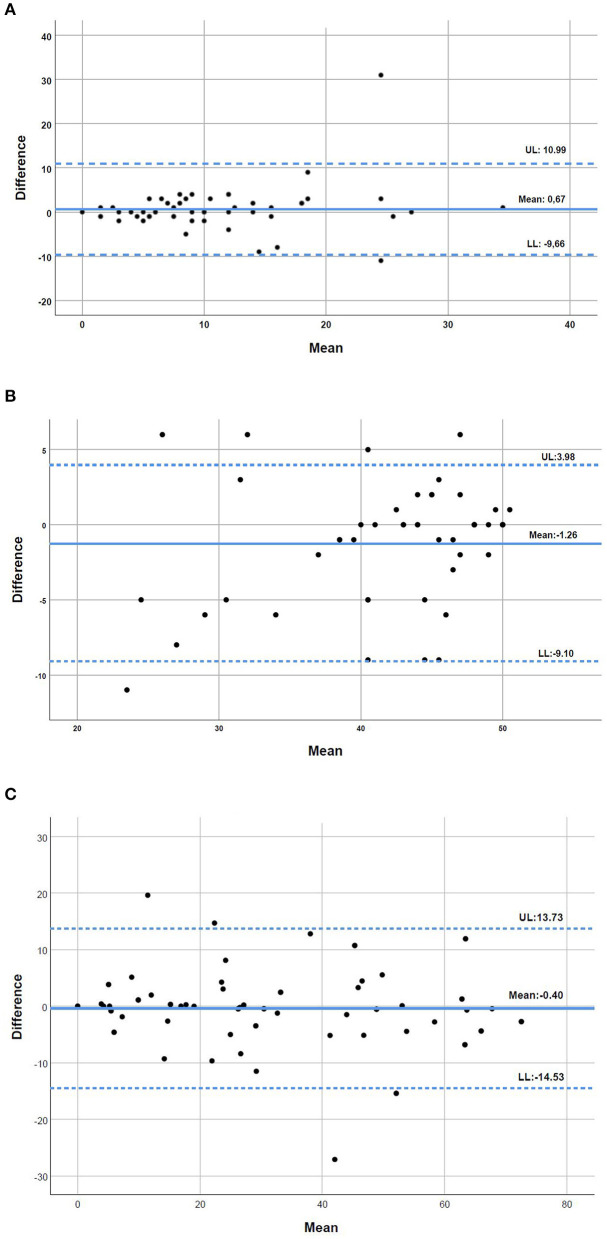
**(A–C)** Bland and Altman plots for the level of agreement between the COPD Assessment Test (CAT), the Functional Assessment of Chronic Illness-Fatigue-Subscale (FACIT-FS) and the St. George's Respiratory Questionnaire (SGRQ) applied in person and by telephone interview to people with chronic obstructive pulmonary disease (*n* = 55). The bold line represents the mean difference in the two types of interview and the dotted lines the 95% upper and lower limits of agreement (UL and LL, respectively).

The Bland and Altman plots illustrated a good level of agreement between the two modes of administration of the questionnaires, with no evidence of systematic bias (Figure 1). The CAT plot showed a mean difference of 0.67 and upper and lower limits of 10.99 and −9.66, respectively (Figure 1A). The FACIT-FS plot illustrated a mean difference of −1.26 and upper and lower limits of 3.98 and −9.10, respectively (Figure 1B). The SGRQ plot showed a mean difference of −0.40 and upper and lower limits of 13.73 and −14.53, respectively (Figure 1C).

### Validity

**Figure 2:**
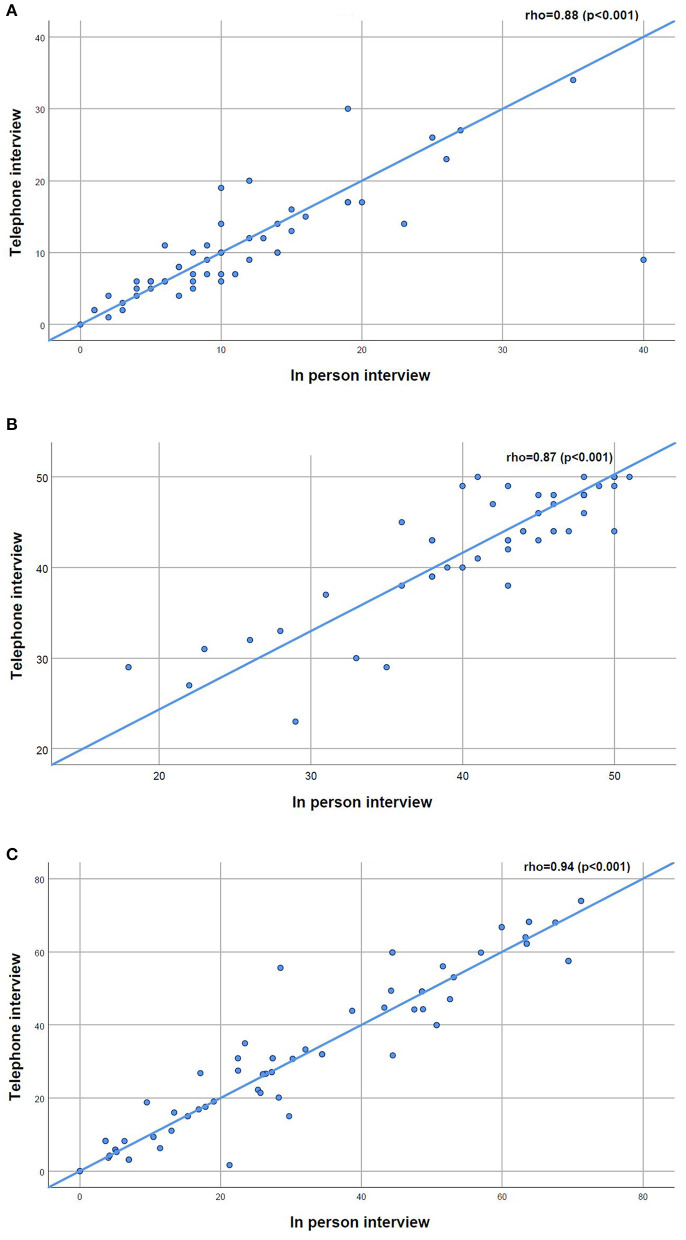
**(A–C)** Scatterplots for the relationship between the COPD Assessment Test (CAT), the Functional Assessment of Chronic Illness-Fatigue-Subscale (FACIT-FS) and the St. George's Respiratory Questionnaire (SGRQ) applied in person and by telephone interview to people with chronic obstructive pulmonary disease (*n* = 55).

Robust positive correlations were found between the total scores of the CAT (rho = 0.88, *p* < 0.001), the FACIT-FS (rho = 0.87, *p* < 0.001) and the SGRQ (rho = 0.94, *p* < 0.001) collected in person vs. by telephone interview (Figure 2). A slightly higher dispersion toward higher values of fatigue was observed in the FACIT-FS plot (Figure 2B).

## Discussion

### Main Findings

Our study shows that the telephonic administration of the CAT, the FACIT-FS and the SGRQ is a reliable and valid alternative approach to in person interviews for monitoring symptoms and HRQoL in people with COPD.

The similar values of the Cronbach's alpha found between the two modes of administration showed their equivalent application. The excellent test–retest reliability, the high agreement and the lack of systematic bias further reinforced the use of telephonic interviews to assess these important PROMs. A strong correlation (>0.70) between the two modes of administration has also been found in all three questionnaires, confirming the validity of telephone interview to assess symptoms and HRQoL in this population (25, 46, 47). These results are in line with prior studies (21, 22) showing that the interview mode (telephone vs. in person) did not influence the health surveys' outcomes. Furthermore, previous evidence (36, 48, 49) demonstrated that the CAT, the FACIT and the SGRQ scores and its psychometric properties do not seem to be influenced by the method of administration. Thus, researchers and clinicians may be confident in applying these questionnaires by telephone, and its use in remote monitoring is encouraged.

The SGRQ showed the greatest reliability and validity when compared to the other two questionnaires, but the subscale of symptoms presented the highest values of associated measurement error. This finding might be explained by the questionnaires' different structure and recall period (33, 36, 38, 50). Specifically in the SGRQ (38) symptoms subscale, questions rely on the presence of cough, sputum, dyspnoea, etc. in the last 3 months and answers are “most days a week,” “several days a week,” “some days a month,” “only with respiratory infections,” or “not at all.” These questions require each participant to report symptoms occurring over a long period of time in contrast to the CAT (33) and the FACIT-FS (36) which refer to the presence and/or frequency of symptoms in the present moment or in the last 7 days, respectively. Evidence suggests that patients tend to value more and consequently overreport more severe symptoms that occurred in the last few days (51, 52). Additionally, it is consensual that symptoms in COPD vary significantly throughout the day and over the weeks and/or months (52, 53). It is, therefore, likely that individuals find difficult to provide a precise report over a 3-months recall period, leading to a higher measurement error.

We also observed that the FACIT-FS showed the higher difference (−1.26) between the in person and telephone interviews compared to the CAT (0.67) and the SGRQ (0.40), as well as a slightly dispersion of values toward higher values of fatigue. A possible explanation might be that the difference between the two modes of administration tended to increase as the scores of the scale increase, suggesting that participants tend to report with more consistency fatigue levels when they were high and caused a perceived higher impact on their daily life. The relatively small sample size and the high symptom variability, confirmed by the results of the SGRQ symptom domain, might also explain our findings. Thus, more research is needed to enhance our knowledge on this and other PROMs, namely exploring the agreement applied by telephone and in person.

The telephone interview might have additional advantages in comparison to in person questionnaires, since it may save costs associated with the participants' transportation, enhance adherence due to the flexibility for scheduling the interview, or improve confidence to speak without having the physical presence of the interviewer (8, 54). These advantages are important in the context of current and future pandemics, but also for researcher planning of observational studies and clinical trials, which may consider to collect some of the information using telephone interview. Nevertheless, in some cases people with COPD might prefer face-to-face contacts which allow a more thorough assessment. Thus, whenever possible the preferences of people with COPD on the mode of assessment should be considered. Additionally, further research exploring these preferences would be important for supporting the use of technologies of information and communication in people with COPD.

### Strengths and Limitations

Strengths of our study include addressing a highly needed topic in the current pandemic, with implications for research and clinical practice, and the random order of the administration modes. Some limitations should also be acknowledged. Our sample size might have influenced our results, since according to the guidelines of the COSMIN initiative (25), a sample size over 100 individuals is desirable for testing reliability and validity of PROMs. Nevertheless, we were able to show the reliability and validity properties of the CAT, the FACIT-FS and the SGRQ including the minimal sample size required (more than 50 individuals), and we could hypothesize that a larger sample would strengthen our results. The interviewer presence applying the CAT and the SGRQ questionnaires, which were originally developed to be self-administered, may be perceived as a possible limitation, since it might bias individuals toward more favorable responses (55). This is however unlikely to have had a significant impact on our results, since the interviewer was present in both modes of administration, thus his/her possible influence was similar in both modes of administration. Moreover, both face-to-face and telephone questionnaires were applied by the same well-trained interviewer to improve consistency. The lack of standardization of the day period in which questionnaires were applied might have influenced our results, since people with COPD tend to report worse symptoms in the morning (56). Further studies should consider to collect this information and standardize the timing of application. Finally, our sample was composed by older adults (from 60 to 76 years old) mainly male and with a low level of education (i.e., ≤ 4 years of education). Thus, participants' characteristics limited external validity to all people with COPD.

### Implications for Research and Clinical Practice

The findings of our study have important implications for research and clinical practice highlighting the added-value of technologies of information and communication to ensure a safe follow-up of meaningful outcomes such as symptoms and health-related quality of life in people with COPD, when face-to-face contact needs to be reduced or is not possible. Moreover, our results also suggest that researchers might consider to collect some baseline and follow-up information using the telephone interview, without compromising the validity and reliability of the results. This is a valuable information in the context of the COVID-19 pandemic, but also in the case of future health outbreaks.

## Conclusion

The telephone interview is a reliable and valid method to assess and monitor symptoms and HRQoL in people with COPD using recommended PROMs, such as the CAT, the FACIT-FS and the SGRQ. The telephone might be an important add-on for personalized assessment and management of chronic respiratory disease in the context of remote monitoring. Nevertheless, the level of adherence and satisfaction of people with COPD with the replacement/addition of these technologies to the standard care should be explored.

## Data Availability Statement

The raw data supporting the conclusions of this article will be made available by the authors, without undue reservation.

## Ethics Statement

The studies involving human participants were reviewed and approved by Centro Hospitalar do Médio Ave reference 09/2016 and 10/2018; Unidade Local de Saúde de Matosinhos reference 10/CES/JAS 17/02/2017 and 73/CE/JAS 12/10/2018; Centro Hospitalar Baixo Vouga reference 777638 and 086892; Hospital Distrital da Figueira da Foz reference 1807/2017 and 27/05/2019; Administração Regional de Saúde do Centro reference 64/2016 and 85/2018. The patients/participants provided their written informed consent to participate in this study.

## Author Contributions

VR, CJ, and AM conceived the study. VR performed data collection, data analysis and interpretation, and drafted the manuscript. AM was responsible for obtaining the funding and ensured the project administration and resources. All authors critically revised the manuscript, ensured accuracy and integrity of the work, approved the final version to be published, and agreed to be accountable for all aspects of the work.

## Funding

This work was supported by Programa Operacional de Competitividade e Internacionalização – POCI, through Fundo Europeu de Desenvolvimento Regional - FEDER (POCI-01-0145-FEDER-028806), by Fundação para a Ciência e Tecnologia (PTDC/SAU-SER/28806/2017), and under the project UIDB/04501/2020.

## Conflict of Interest

The authors declare that the research was conducted in the absence of any commercial or financial relationships that could be construed as a potential conflict of interest.

## Publisher's Note

All claims expressed in this article are solely those of the authors and do not necessarily represent those of their affiliated organizations, or those of the publisher, the editors and the reviewers. Any product that may be evaluated in this article, or claim that may be made by its manufacturer, is not guaranteed or endorsed by the publisher.
